# How scientific research reacts to international public health emergencies: a global analysis of response patterns

**DOI:** 10.1007/s11192-020-03531-4

**Published:** 2020-06-09

**Authors:** Lin Zhang, Wenjing Zhao, Beibei Sun, Ying Huang, Wolfgang Glänzel

**Affiliations:** 1 School of Information Management, Wuhan University, Wuhan, China; 2 Centre for R&D Monitoring (ECOOM) and Department of MSI, KU Leuven, Leuven, Belgium; 3 Department Science Policy and Scientometrics, Library of the Hungarian Academy of Sciences, Budapest, Hungary

**Keywords:** PHEIC, COVID-19, Response pattern, Bibliometrics, Scientometrics

## Abstract

As of the middle of April 2020, the unprecedented COVID-19 pandemic has claimed more than 137,000 lives (https://coronavirus.jhu.edu/map.html). Because of its extremely fast spreading, the attention of the global scientific community is now focusing on slowing down, containing and finally stopping the spread of this disease. This requires the concerted action of researchers and practitioners of many related fields, raising, as always in such situations the question, of what kind of research has to be conducted, what are the priorities, how has research to be coordinated and who needs to be involved. In other words, what are the characteristics of the response of the global research community on the challenge? In the present paper, we attempt to characterise, quantify and measure the response of academia to international public health emergencies in a comparative bibliometric study of multiple outbreaks. In addition, we provide a preliminary review of the global research effort regarding the defeat of the COVID-19 pandemic. From our analysis of six infectious disease outbreaks since 2000, including COVID-19, we find that academia always responded quickly to public health emergencies with a sharp increase in the number of publications immediately following the declaration of an outbreak by the WHO. In general, countries/regions place emphasis on epidemics in their own region, but Europe and North America are also concerned with outbreaks in other, developed and less developed areas through conducting intensive collaborative research with the core countries/regions of the outbreak, such as in the case of Ebola in Africa. Researches in the fields of virology, infectious diseases and immunology are the most active, and we identified two characteristic patterns in global science distinguishing research in Europe and America that is more focused on public health from that conducted in China and Japan with more emphasis on biomedical research and clinical pharmacy, respectively. Universities contribute slightly less than half to the global research output, and the vast majority of research funding originates from the public sector. Our findings on how academia responds to emergencies could be beneficial to decision-makers in research and health policy in creating and adjusting anti-epidemic/-pandemic strategies.

## Introduction

In April 2020, we are in the midst of the COVID-19 pandemic, the spreading of which does not yet show any sign of success in flattening the infection curve. Italy and Spain are in their darkest hours, and the impending devastation in the USA looms alarming news breaks each hour. The attention of the global scientific community has turned toward flattening the curve, stopping the spread, treating the infected patients and racing to find a vaccine. However, time is short and, as governments pump billions of dollars into keeping their economies afloat, money is even shorter. This makes an international review of what research is conducted, where and by whom research is done, an insightful exercise and useful source of an expeditious and efficient global research effort.

In situations like this, when the global research landscape is changing every day, it would be an unfeasible and even irresponsible attempt to closely follow up research and to keep the review up to date. What may be more valuable is to identify the ‘response patterns’ of academia in the face of public health emergencies towards controlling and stopping outbreaks. Therefore, we aim to achieve two goals in this paper. The first one is to provide insights into how academia responds to the pandemic situation through a comprehensive analysis of over 32,000 articles and reviews on five virus outbreaks during the last 2 decades. The exercise comprises the volume of research, geopolitical region, subject field, research sector and funding agency. The second goal is to provide a preliminary review of the global research effort in defeating COVID-19 in the light of the response patterns detected in the first part of the analysis. These insights into the characteristics of research output in the relevant domains could assist policymakers in planning, creating and adjusting scientific strategies for a preferable response to health emergencies.

Public health emergencies confronting society have by nature strong science and technology components, which means that substantial improvements in health come as a result of significant advances in knowledge and technology (Howitt et al. 2012). Hence today, controlling pandemics rests to the largest extent in the hands of scientific researchers—first, by understanding the sources and transmission routes of the infection, then through ways to reduce infection rates and finally with the development of treatments, cures and methods of prevention. The most common outlet for scholars to communicate and fight through each of these stages is via academic publications (Persson et al. 1997). Researchers are producing publishable work in record time (Chen et al. 2020; Holshue et al. 2020; Li et al. 2020; Lu et al. 2020). Journals are slashing the typical time lags between peer review and publication, processed within days, what otherwise might have taken months or even years. Funding agencies have created special grants supporting immediate research on COVID-19 (Medical Research Council 2020; National Natural Science Foundation of China 2020).

Using bibliometric approaches to analyse scientific output relating to recent public health emergencies helps reveal the specific contributions academia makes to resolve health crises. Several studies stand out as good examples of where bibliometrics has provided a useful international view of medical and multidisciplinary research efforts. For example, a global research of Fabry’s disease conducted by Klingelhöfer et al. (2020) identified the need to foster research infrastructure on this rare disease in developing countries with the focus on genetic research. Other studies include exploring the research status and directions on several infectious diseases, such as tuberculosis (Ramos et al. 2008), leishmaniasis (Soosaraei et al. 2018), and leprosy (Khasseh et al. 2016). Several studies of emergent infectious diseases have been conducted on single outbreaks in specific countries/regions. Those studies relied on research collaboration, publication language and citation impact to map the publication-activity patterns and scholarly impact of research related to the diseases (Chiu et al. 2004; Luchs 2012; Nasir and Ahmed 2018; Pouris and Ho 2016). As far as we know, the present paper presents the first comparative bibliometric study of multiple outbreaks in a global context that has ever been conducted.

The main research questions addressed in this paper are as follows:

1. Is there any identifiable response from academia to public health emergencies? If so, what are the features of publication dynamics and distribution by country/region and research discipline?
2. What kinds of research institutions and funding agencies are the main actors in research relating to public health emergencies?
3. How has academia, and especially Chinese academia, reacted to the early stages of the current COVID-19 pandemic?

The paper unfolds as follows. The next section provides a brief background on COVID-19, public health emergencies according to the WHO, and our strategy for collecting publication data on recent infectious disease outbreaks. In “Results: the first group—Zika, H1N1, Ebola and SARS” section, we present the patterns and participants on the previous outbreaks, followed by the preliminary analysis and discussion on COVID-19 in “Preliminary analysis: COVID-19” section. The last section contains our findings and reflections on this research, plus our intended directions for future work.

## Background and data collection

COVID-19 is an infectious disease caused by a newly discovered coronavirus, which was firstly reported in December 2019. The disease rapidly spread with a continued increasing number of confirmed cases and, by 30 Jan 2020, the World Health Organization (WHO) had proclaimed COVID-19 to be a Public Health Emergency of International Concern (a PHEIC). A PHEIC is a formal declaration by the WHO of “an extraordinary event which is determined to constitute a public health risk to other States through the international spread of disease and to potentially require a coordinated international response”.^1^ PHEICs were instituted following the SARS epidemic of 2002–2003 as a way of building surveillance and early warning systems for outbreaks, mobilising rapid international responses and containing the spread of infection at the source (Hoffman 2010; WHO 2005). The twenty-first century has seen six PHEIC declarations since SARS: the 2009 H1N1 pandemic, the 2014 Polio declaration, the 2014 outbreak of Ebola in West Africa, the 2015–2016 Zika virus epidemic, the 2018–2020 Kivu Ebola epidemic, and the 2019–2020 coronavirus outbreak.

Given our aim to map the patterns of academic ‘response’ to severe emergencies, we deemed all these outbreaks to be primary research subjects, with the exception of polio. This is because PHEIC warns of polio’s resurgence after its near-eradication. Hence, including literature on a virus that has been of significant concern to academia for over a century and to the WHO since its inception would introduce considerable bias into the sample. Details of SARS and the five subsequent PHEICs included in the study are provided in Table 1.

**Table 1:** Information on SARS and five public health emergencies of international concern (PHEIC)

Outbreak duration	PHEIC announced	Country/region	Epidemic
Dec 2002–July 2003	n/a	China, Southeast Asia	Severe Acute Respiratory Syndrome (SARS)
Mar 2009–Aug 2010	Apr 2009	Mexico, the USA, etc.	influenza A(H1N1)
Dec 2013–Jan 2016	Aug 2014	West Africa, Spain, etc.	Ebola
Apr 2015–Nov 2016	Feb 2016	Brazil, Caribbean	Zika
Aug 2018–Present	Jul 2019	DR Congo	Ebola
Dec 2019–Present	Jan 2020	Worldwide	COVID-19

We subdivided these viruses presented in Table 1 into the first four viruses and COVID-19, and analysed the two groups separately. The analysis of COVID-19 is only preliminary due to the fast-developing situation currently. The document retrieval strategies for the first four viruses, as shown in Table 2, are constructed by referring to entry terms of each specific name of virus/disease in the MeSH vocabulary^2^ and retrieving rules of WoS. Our final sample of the first four viruses, drawn from the Science Citation Index Expanded (SCI-E) & Social Sciences Citation Index (SSCI) of the Clarivate Analytics’s Web of Science (WoS) Core Collection, comprised 32,167 articles and reviews. Note, however, that the number of publications in Table 2 will add up to more than total since some publications related to more than one disease.

**Table 2:** Search strategy for publications on four infectious diseases from WoS

Virus	Search strategy	No. of publications
Zika	TS = (“ZikV” OR “Zika”)	5428
H1N1	TS = (“H1N1”)	15,409
Ebola	TS = (“Ebola*”)	6489
SARS	TS = ((“Severe Acute Respiratory Syndrome”) OR “SARS-CoV” OR ((Coronavirus OR Virus) AND SARS))	5685

Because of the limited number of publications indexed by WoS in the initial stage of the outbreak, we expanded our search for publications on COVID-19 beyond WoS to conduct a more comprehensive analysis. We retrieved articles and reviews in PubMed, and the source categories of the Chinese Social Sciences Citation Index (CSSCI) and Chinese Science Citation Database (CSCD) in CNKI (Chinese National Knowledge Infrastructure), which is the largest continuously updated database of Chinese journals in the world. The search strategies are listed in Table 3. Data were obtained on 9 April 2020. All retrieved publications were manually checked, and irrelevant publications were removed. The final corpus comprised of 3069 publications.

**Table 3:** Search strategies for publications on COVID-19

Database	Search strategy	No. of publications
WoS	TS = (“2019-nCov” OR “2019 novel coronaviru*” OR “2019 novel-coV” OR “COVID-19” OR “SARS-CoV-2”)	231
PubMed	Search ((((“2019-nCov”) OR “novel coronaviru”) OR “2019 novel-coV”) OR “COVID-19”) OR “SARS-CoV-2”	2170
CNKI	Search multiple expressions of COVID-19 and related terms in both Chinese and English in Title/Abstract/Keywords	668

## Results: the first group—Zika, H1N1, Ebola and SARS

### Publication dynamics

**Fig. 1:**
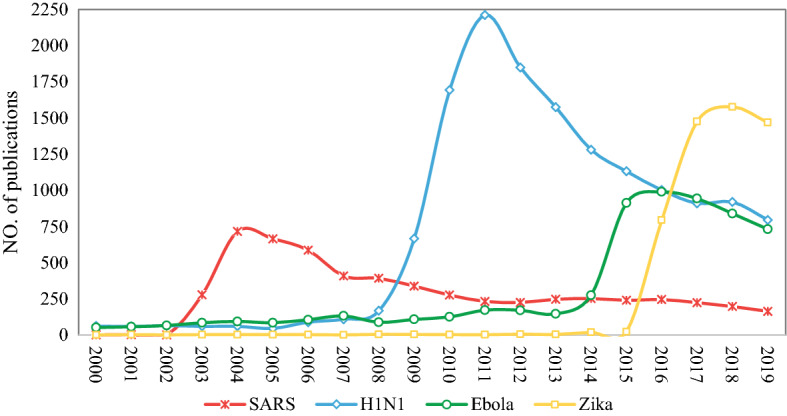
Number of publications on the four infectious diseases by year (2000–2019)

Our first observation is that very few publications on any of these viruses were published prior to 2000. Hence, Fig. 1 shows the number of publications by year between 2000 and 2019. From all four cases, a clear trend is apparent. Immediately following the outbreak, there is a sharp rise in disease-specific publications up to a peak within 2 years after the outbreak, beyond which the number of publications gradually decreases. These patterns reflect a quick and remarkable response by the research community to public health needs.

SARS was first reported in China’s Guangdong Province in November 2002 and was recognised by the WHO at the end of February 2003. The first study on SARS appeared in 2003, and the number of publications peaked at 716 in 2004. There have not been known reports of SARS infection since 2004. Research interest decreased with the end of the SARS outbreak resulting in a clearly perceptible and continuous decline of publication activity in SARS-related research during the last 15 years.

Accounting for almost half the total number of publications, more researches have been published about H1N1 than on any other. The 2009 H1N1 influenza pandemic, which lasted from early 2009 to late 2010 (Carmo and Oliveira 2009), was first detected in the USA and spread quickly across the country and in all other regions of the world. Research on H1N1 reached its climax in 2011 with as many as 2200 publications.

Ebola Virus Disease (EVD, or simply Ebola) is a rare and deadly disease of human and other primates that typically occurs in the tropical regions of sub-Saharan Africa (WHO 2020a). Corresponding with the first known Ebola outbreak in 1976 in South Sudan and DR Congo, the first scientific article related to Ebola in our database was published in 1977. However, academic attention to Ebola did not increase significantly until a massive Ebola epidemic broke out in 2014 in West Africa and Nigeria amid fears of catastrophe to the rest of the world (Cowell 2014). The Director-General of the WHO declared the Ebola PHEIC on 8 August 2014 (WHO 2014), and a clear upward trend in publication activity on Ebola ensued lasting until 2016. A further Ebola PHEIC was declared in 2018 as a result of renewed emergence in DR Congo, but this following announcement did not result in any perceptible increase in publication activity. In fact, the number of publications on Ebola, which was already in decline since 2016, experienced an even slightly sharper drop.

The Zika virus first isolated from a rhesus monkey in the Ugandan Zika Forest in 1947, later from Aedes africanus mosquitoes in the same forest (Nasir and Ahmed 2018). From the 1960s to the 1980s, rare sporadic cases of human infections were found across Africa and Asia. Research on Zika appeared early in our database, but publication numbers remained low until the outbreak in 2015–2016, which resulted in sharply growing attention with a peak in 2018.

### International comparison of national research effort

The ground zero of each outbreak varies as does the research capability of different countries/regions, both affecting the geographic distribution of research effort. The country/region assignment of publications has been done on the basis of the author’s institutional address as reported in the by-line of the paper. In this study, a full-count assignment scheme has been applied, that is, national publication counts express how many papers the country/region has contributed to.

**Fig. 2:**
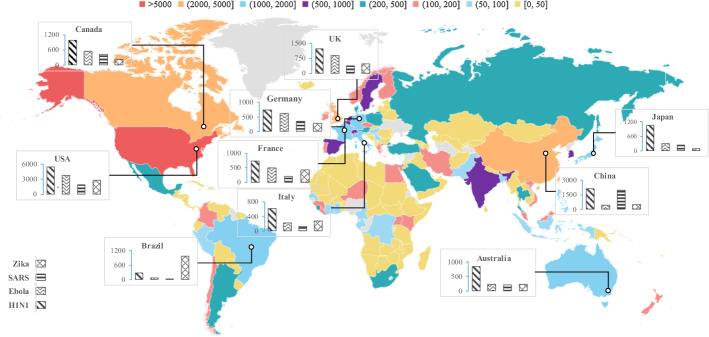
The geographical distribution of publications on the four infectious diseases—top 10 countries/regions by number of publications

Figure 2 shows the geographical distribution of publications for the top 10 countries**/**regions by the number of publications on the four viruses. The USA, China and the UK are the three countries with the largest number of publications. The USA ranks first in all viruses except SARS. Within each disease, the distribution patterns across Europe and North America are quite similar, i.e., a relatively higher proportion of publications on H1N1 and Ebola than for Zika and SARS. H1N1 got lots of exposure from all ten countries except for Brazil, which has devoted most of its effort to Zika. In addition, studies on H1N1 are relatively evenly distributed, whereas papers on the other three diseases show significant regional differences. The three countries with the most publications on Ebola are the USA, UK and Germany, even though both Ebola outbreaks had their epicentre in Africa. Unlike Ebola, the country with the most SARS-related publications was the one in the heart of the outbreak—China.

In general, these ten countries tended to place greater emphasis on diseases that caused epidemics in their own regions, especially SARS in China and Zika in Brazil. Countries in Europe and North America paid relatively close attention to Ebola, which was not an epidemic in their own regions, perhaps partially because of the high fatality rate coupled with early media reports of isolated cases in the UK (Cooper 2014) and the USA (Botelho and Wilson 2014) that eventually did not escalate.

**Fig. 3:**
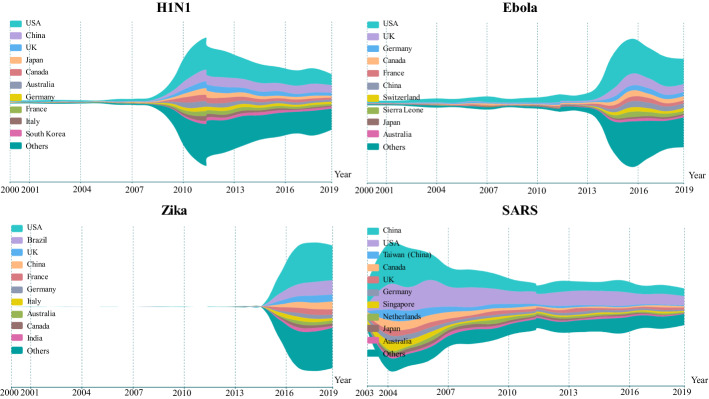
Number of publications on the four infectious diseases according to countries/regions by year

We further analysed the geographical distributions of publications by year to explore the differences in response speed and continuity of research from various countries/regions. Figure 3 shows the variations of publication distributions with the top 10 countries/regions from 2000 to 2019, where the vertical axis refers to the number of publications for each disease. The top 10 countries/regions that contributed to the highest number of publications vary for four diseases, in which the USA, the UK, China, Canada, Australia and Germany are on the four diseases’ top 10 list. Interestingly, Chinese researchers showed a strong and quick response to the SARS outbreak in 2003–2004. However, the research interest declines steadily with the end of the outbreak. In contrast to China, the USA tends to pay more sustained attention to SARS. Of particular note is that Sierra Leone is the only African country that appeared on the list of Ebola, which is the core area of the 2014 outbreak of Ebola in Western Africa (WHO 2014).

**Fig. 4:**
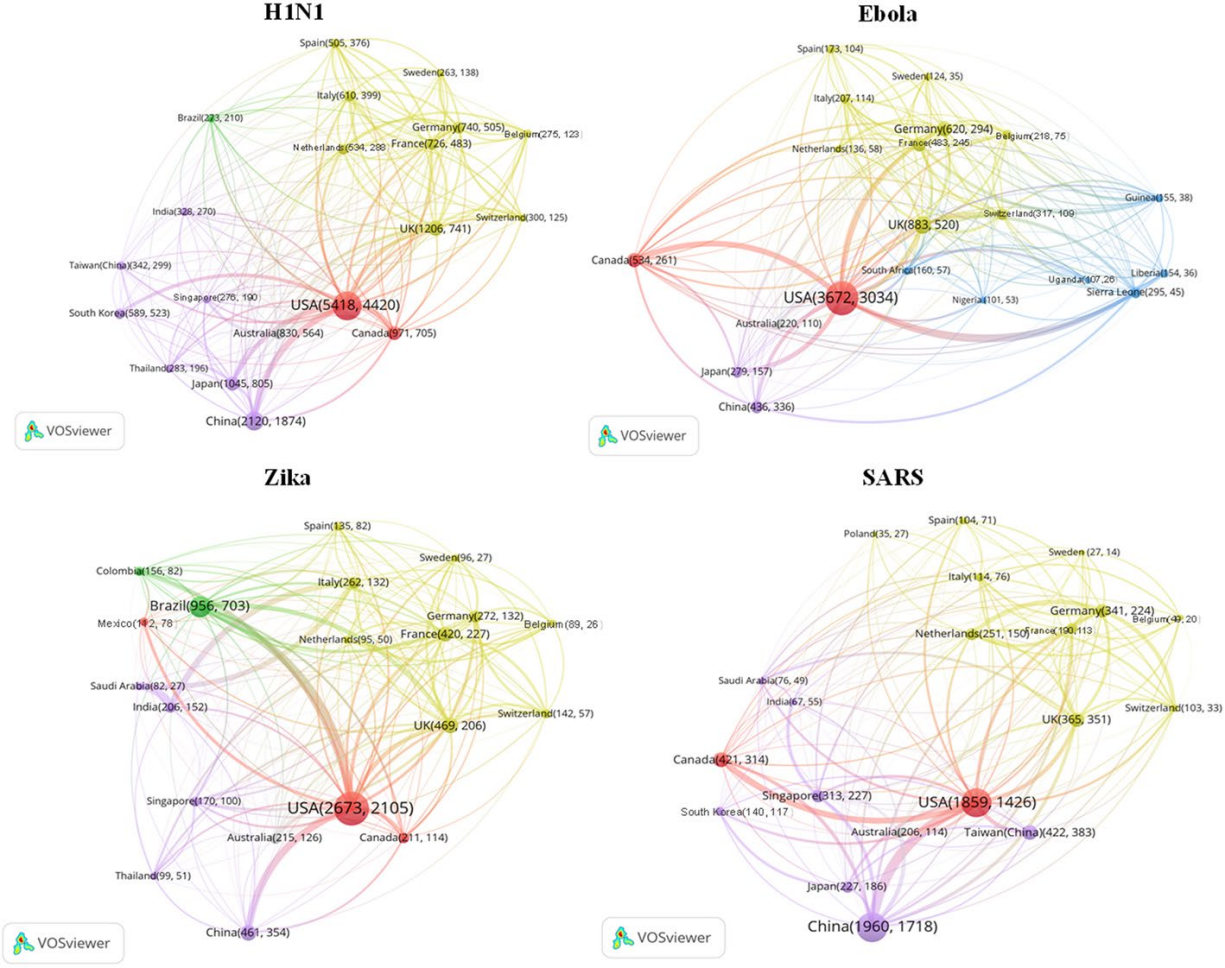
International collaboration patterns of publications on the four infectious diseases

To further investigate joint research efforts by different countries/regions on health emergencies, we generated international collaboration networks among the top twenty countries/regions according to their publication activity of four viruses (see Fig. 4). The size of a node indicates the total number of publications of the country/region, which corresponds to the first number presented on the node. The second number on each node represents the number of papers published by the country/region as the first authors’ address. The thickness of links refers to the strength of collaboration between two countries/regions. It is calculated based on Salton’s measure, which is defined as the number of joint publications divided by the square root of the product of the number (i.e., the geometric mean) of total publication outputs of the two countries. The different colours of links and nodes refer to the continent on which the country/region is located.

With the largest number of publications in total, the USA plays a significant role in collaboration with other countries/regions regarding all these four diseases. Apart from Canada and European countries, the USA also collaborated intensively with several African countries on Ebola research, such as Sierra Leone, Liberia, Guinea etc., which are the main areas of the Ebola outbreak. African countries, although particularly contributed to Ebola research, in general, do not play a leading role in international collaborations, which is also reflected by the low share of ‘first-author’ publications. Different from the massive joint efforts contributed by the USA and European countries on Ebola, SARS related research reveals a quite different pattern that European countries had a relatively weak role in this China-centric outbreak. China, as the country with the largest number of publications in SARS, focussed more on collaboration with the USA.

### Disciplinary analysis

**Fig. 5:**
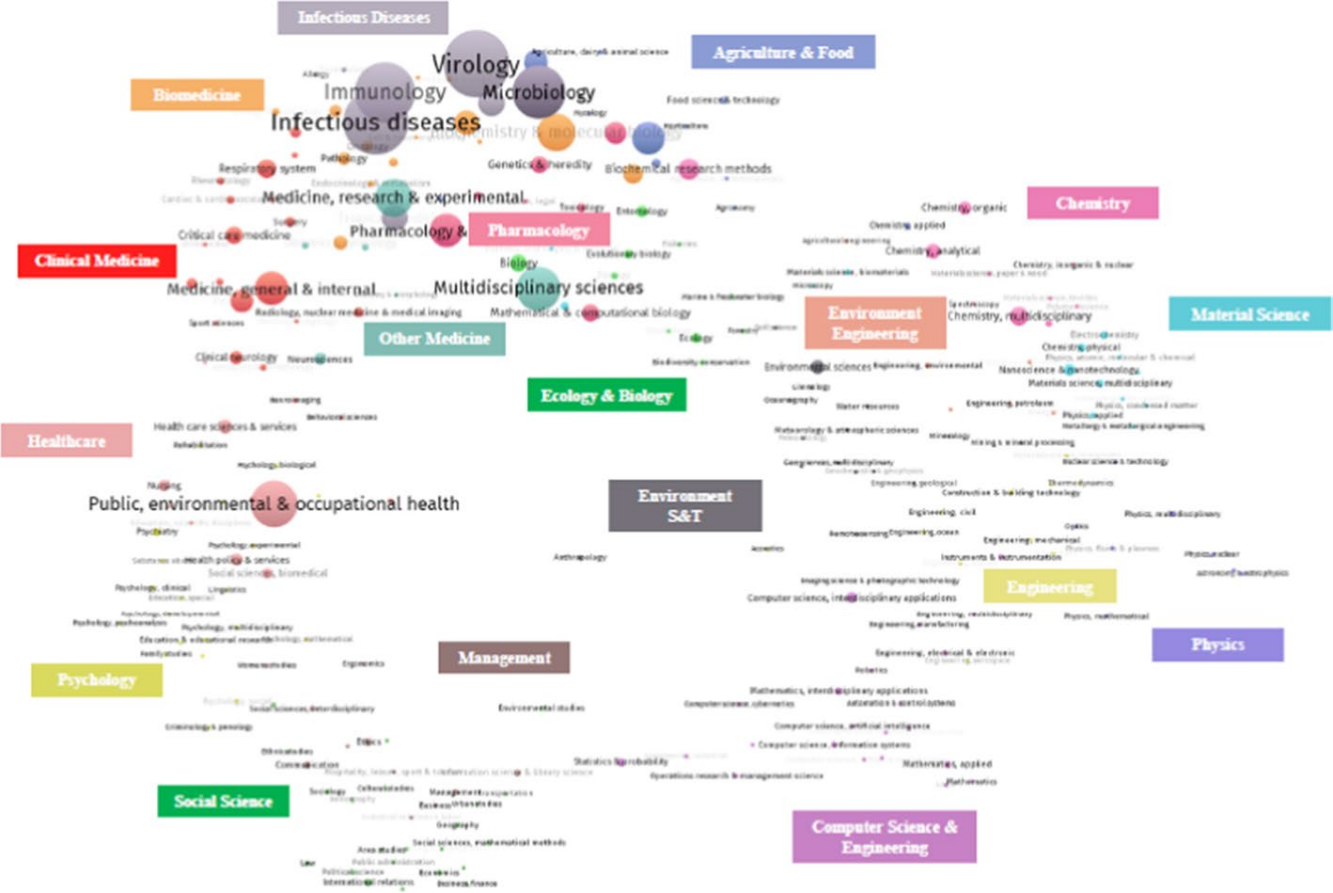
The science overlay map of the publications on the four infectious diseases [*Note* The base map of discipline was developed from the matrix of 227 × 227 cells of WoS categories, which generated on the basis of direct citation counting and normalised with the cosine function (Carley et al. 2017)]

In this section, we adopt the science overlay maps proposed by Rafols et al. (2010) and Carley et al. (2017), and generated using the VOSviewer (Waltman et al. 2010) to illustrate those disciplines that proved most relevant for research on these four infectious diseases, shown as Fig. 5. Every node presents a WoS category, and the size of the node indicates the number of the publications based on a full counting scheme.

As Fig. 5 shows, publications have a relatively centralised distribution around those Subject Categories, which primarily belong to medical and health-related fields, such as ‘Infectious Diseases’, ‘Health Care’, ‘Pharmacology’ and ‘Clinical Medicine’. A non-negligible share of papers has also been published outside the core of the life sciences, above all in ‘Chemistry’, ‘Environmental Science’ and ‘Material Science’—fields with relevant links to medical research.

**Fig. 6:**
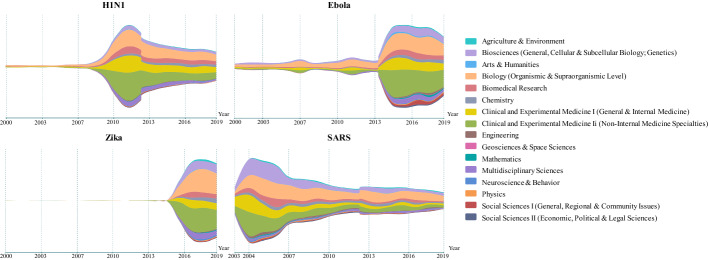
Number of publications on the four infectious diseases according to the 16 ECOOM major fields by year

The structure of the fine-grained picture base on the WoS Subject Categories provided in Fig. 5 is strongly influenced by multiple subject assignments, on the one hand, and the different broadness of the journals’ scopes underlying this assignment, on the other hand. Therefore, we further used the 16 major fields of the ECOOM classification scheme (cf. Glänzel and Schubert 2003; Glänzel et al. 2016), which actually forms an aggregation of the WoS categories, to produce a more explicit depiction of the subject distribution. The charts in Fig. 6 show the annual change of this distribution, where the vertical axis represents the number of publications.

‘Clinical and Experimental Medicine I (Internal Medicine)’, ‘Clinical and Experimental Medicine II (Non-internal Medicine)’, ‘Biology’ and ‘Biosciences’ are the four subject fields with the most publications on all four diseases. The remarkable attention given to Ebola in ‘Social Science I’ (with regional and community health issues) is worth mentioning as it certainly reflects the social perspectives of this epidemic. According to the WHO (2020a), “Ebola is rare, but the disease has a high risk of death, killing 25% to 90% of those infected”. The symptoms of the Ebola virus can be sudden and striking, including high fever, fatigue, vomiting and both internal and external bleeding. The high mortality rate and scary symptoms have led to public panic about Ebola. Furthermore, the generally lower level of economic and technological development in the affected areas of Africa could be another reason for the severe societal problems resulting from the epidemic and the close attention given to this disease by social sciences as well.

SARS coronavirus (SARS-CoV) was identified and officially declared an epidemic in 2003. Corresponding to the need for both a fundamental understanding of the virus and clinical treatments of the disease, research studies of SARS were distributed more evenly over ‘Clinical and Experimental Medicine I (Internal Medicine)’, ‘Clinical and Experimental Medicine II (Non-internal Medicine)’, ‘Biosciences’ and ‘Biology’. Towards the end of the outbreak, there was a decreasing demand for treatments; hence, the higher share of publications in ‘Biology’.

**Fig. 7:**
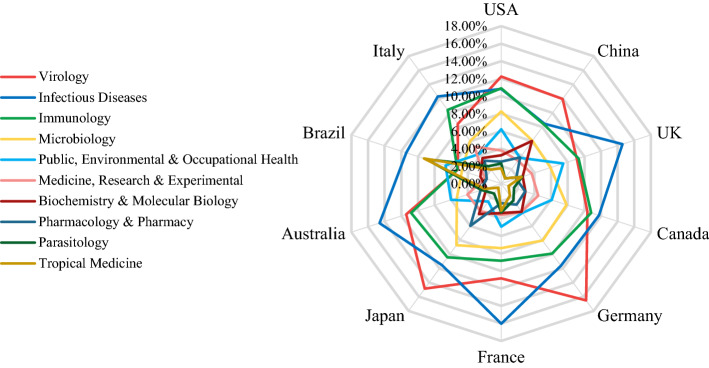
The radar map of discipline proportion for publications on the four infectious diseases of top 10 countries

In order to gain a more in-depth insight, we combined geographical distributions with the disciplinary one. The above analysis indicated quite similar patterns in the disciplinary distributions of publications in general. In a next step, we subdivided the major fields into the ten most frequent WoS Subject Categories in medical science for a fine-grained exploration of the differences between countries/regions. Thus, instead of the bottom-up approach in Fig. 5, we applied a top-down solution to filter out redundancies caused by multi-assignment and journals with ‘general scopes’ and to focus on the most relevant disciplines. Figure 7 illustrates the results, where the ten radial lines depict the discipline distributions of ten countries’ publications on the listed fields, and edges with different diameters of the map represent the percentage of publications involved by each country in the listed fields. For example, almost 15% of publications that Australia contributed to was devoted to the category ‘Infectious Diseases’.

As shown in Fig. 7, ‘Virology’, ‘Infectious Diseases’ and ‘Immunology’ are the top three categories with the largest shares of publications in all countries except for Brazil. As the centre of the Zika outbreak, Brazil devoted the lion’s share of its research efforts to this virus as compared with the other three. Zika is primarily transmitted by the Aedes mosquito, which makes it highly relevant to ‘Parasitology’, ‘Tropical Medicine’ and ‘Infectious Diseases’—the top three disciplines in Brazilian research. It is particularly noteworthy that Brazil also paid relatively close attention to ‘Public, Environmental & Occupational Health’.

China and Japan also demonstrated quite different disciplinary interests compared to other countries. ‘Tropical Medicine’ is the least studied discipline in China and Japan, followed by ‘Parasitology’. Compared with other European and American countries, ‘Public, Environmental & Occupational Health’ also received less attention from these two Asian countries. It is also interesting that China concentrates more on ‘Biochemistry & Molecular Biology’, while Japan places more emphasis on ‘Pharmacology & Pharmacy’.

To consolidate our observations, we calculated the cosine similarities between disciplinary distribution in different countries. The results in Table 4 confirm our findings that Brazil has distinct differences, and China and Japan have low similarity with the other countries. Although Germany is somewhat similar to Japan, mainly due to sharing the same top four disciplines and relatively much attention paid to ‘Pharmacology & Pharmacy’ (see Fig. 7). Research interests in the USA and Canada are the most similar at the cosine level of 0.988. Australia, the UK and France form kind of cluster with an average cosine similarity of 0.984. Apart from the top three disciplines for these three countries, ‘Public, Environmental & Occupational Health’ also received relatively much attention, above all in the UK.

**Table 4:**
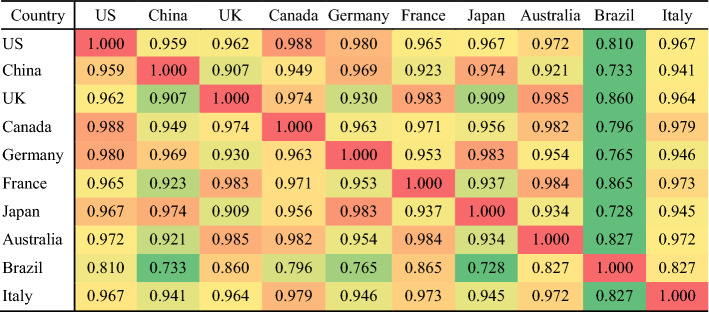
Cosine similarities between the disciplinary distribution of the 10 countries with the most publications on the four infectious diseases

### Institutional research efforts

**Fig. 8:**
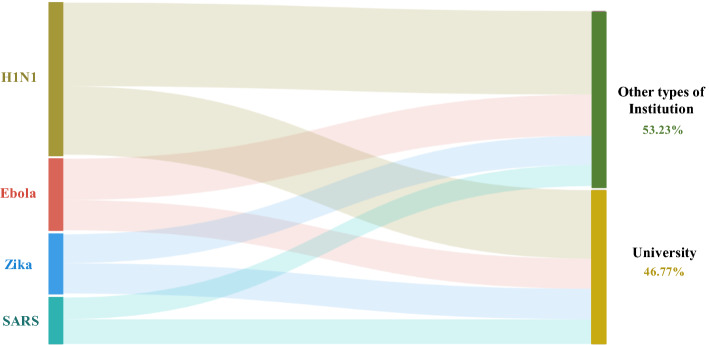
Sankey diagram of publications on the four infectious diseases by research institutions

Scientific output represents not only the efforts of researchers but also the involvement and support of research institutions and funding agencies. The institution information was cleaned through correcting the clerical errors from authors, merging spelling differences and various expressions in different times of institutions. Following the rules of country/region and subject assignment, the number of publications for each research institution was calculated on the basis of a full counting scheme. As expected, universities play a pivotal role in enabling and conducting relevant research and responding to public health emergencies. As shown in Fig. 8, universities have contributed to more than 45% of the publications, which is almost equal to the contribution of all other institutional sectors of research combined.

Table 5 lists the ten institutions with the largest number of publications. Universities and governmental research institutes are the most actively involved in responding to health emergencies. The Centers for Disease Control and Prevention (CDC) and the National Institute of Allergy and Infectious Diseases (NIAID) in the USA have notably contributed to improving understanding of different diseases, especially in the context of H1N1 and Ebola.

**Table 5:** The 10 institutions with the highest number of publications on the four infectious diseases

Institution	Country/region	Institution Type	Institution	Country/region	Institution Type
*SARS*			*H1N1*		
Univ Hong Kong	China	University	Ctr Dis Control & Prevent	USA	Government Agency
Chinese Acad Sci	China	Government Agency	Univ Hong Kong	China	University
Chinese Univ Hong Kong	China	University	St Jude Children’s Res Hosp	USA	Hospital
Ctr Dis Control & Prevent	USA	Government Agency	Univ Toronto	Canada	University
Univ N Carolina	USA	University	Emory Univ	USA	University
Univ Toronto	Canada	University	Univ Melbourne	Australia	University
Natl Taiwan Univ	Taiwan (China)	University	Chinese Acad Sci	China	Government Agency
Natl Univ Singapore	Singapore	University	Harvard Univ	USA	University
Peking Univ	China	University	NIAID	USA	Government Agency
NIAID	USA	Government Agency	Univ Wisconsin	USA	University
*Ebola*			*Zika*		
NIAID	USA	Government Agency	Univ Sao Paulo	Brazil	University
Ctr Dis Control & Prevent	USA	Government Agency	Ctr Dis Control & Prevent	USA	Government Agency
Med Res Inst Infect Dis	USA	Government Agency	Inst Pasteur	France	Non-profit private institution
Publ Hlth Agcy Canada	Canada	Government Agency	Univ Texas Med Branch	USA	University
Univ Manitoba	Canada	University	Fundacao Oswaldo Cruz	Brazil	Government Agency
WHO	–	UN Agency	Chinese Acad Sci	China	Government Agency
Univ Texas Med Branch	USA	University	Univ Oxford	UK	University
Univ Marburg	Germany	University	Emory Univ	USA	University
Univ Penn	USA	University	Univ Fed Rio de Janeiro	Brazil	University
US Army	USA	Government Agency	Univ Florida	USA	University

Furthermore, five of the ten institutions that published research results on Ebola are government agencies in the USA and Canada, which reflect the concern from the developed countries’ government bodies on epidemics in underdeveloped areas. The University of Hong Kong and the Chinese Academy of Sciences are found as the two most active institutions from China. Their focus was more on SARS and H1N1. Consistent with the previous analyses, research institutions in Brazil showed a distinct tendency toward Zika.

### Funding agency

**Fig. 9:**
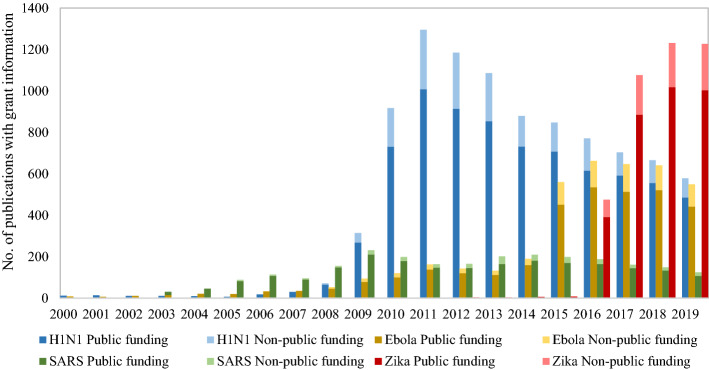
Number of publications on the four infectious diseases with grant information

Funding information in scientific publications often lacks standards and is incomplete. Completeness and accuracy of funding information in bibliographic databases do practically not allow any profound bibliometric analysis at this level (Alvarez-Bornstein et al. 2017; Liu et al. 2020). Nevertheless, to gain some insight into the funding patterns of research on the epidemics, we also analysed the information obtained from the WoS metadata. We found in terms of funding agencies that 19,780 papers (61.49%) in our dataset acknowledged support from a grant, of which 17,307 (53.80%) supplied valid information about the funding agency(s). We have analysed these papers in the following. The assignment of publications with multiple funding agencies is based on full-counting method. We subdivided the funding agencies into public and non-public agencies. Public agencies mainly refer to governmental funding bodies and institutions, including public universities. The non-public sector includes non-profit, charity foundations, private firms and other non-public. The number of publications with grant information by virus is shown in Fig. 9.

H1N1 has the largest number of publications with valid grant information; SARS has the smallest (cf. Figure 9). The SARS outbreak lasted only 18 months with a peak of infection even much before, which might explain why there are so few funded publications for this case. This was a very short period to establish grants and parameters, apply for and receive funding, let alone to have sufficient time for doing research and to publish. Furthermore, research interest declined with the disappearance of the SARS virus. The same seems to hold for funding interest.

**Fig. 10:**
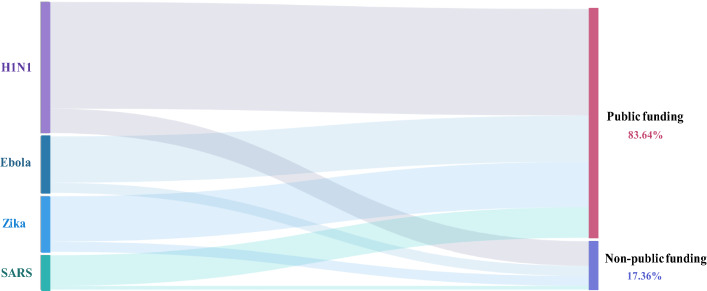
Sankey diagram of publications on the four infectious diseases by funding sectors

Figure 10 shows the breakdown between public and non-public funding. Public funding agencies contributed to more than 80% of the research output for all four viruses, which demonstrates the large degree of governmental support for research in response to public health emergencies.

The public sector dominates the top ten funding agencies. As Table 6 indicates, the USA Department of Health & Human Services (HHS) ranks first in supporting research in all four cases, within which the National Institution of Health (NIH), as one of the world’s leading public medical research and funding organizations, plays a prominent part. The National Natural Science Foundation of China (NSFC) and the European Commission (EU) also made notable contributions to supporting research and, like the other countries/regions, public funding agencies in Brazil occupied an essential position in responding to Zika.

**Table 6:** The 10 funding agencies with the highest number of publications on the four infectious diseases

Funding agencies	Country/region	Agency type	Funding agencies	Country/region	Agency type
*SARS*			*H1N1*		
Dept of Health & Human Services	USA	Public	Dept of Health & Human Services	USA	Public
National Natural Science Foundation of China	China	Public	National Natural Science Foundation of China	China	Public
Ministry of Science and Technology	China	Public	Ministry of Science and Technology	China	Public
European Commission	EU	Public	GlaxoSmithKline	UK	Non-public
Chinese University of Hong Kong	China	Public	Canadian Institutes of Health Research	Canada	Public
National Science Council of Taiwan	Taiwan (China)	Public	European Commission	EU	Public
Food and Health Bureau	China	Public	Medical Research Council	UK	Public
Public Health Service	USA	Public	Ministry of Education, Culture, Sports, Science and Technology	Japan	Public
German Research Foundation	Germany	Public	Istituto Pasteur Italia Fondazione Cenci Bolognetti	Italy	Non-public
Ministry of Education, Culture, Sports, Science and Technology	Japan	Public	Roche, Inc.	Switzerland	Non-public
*Zika*			*Ebola*		
Dept of Health & Human Services	USA	Public	Dept of Health & Human Services	USA	Public
National Council for Scientific and Technological Development	Brazil	Public	Dept of Defense	USA	Public
European Commission	EU	Public	European Commission	EU	Public
Coordenação de Aperfeiçoamento de Pessoal de Nível Superior	Brazil	Public	National Natural Science Foundation of China	China	Public
National Natural Science Foundation of China	China	Public	National Science Foundation	USA	Public
Ministry of Science and Technology	China	Public	Wellcome Trust	UK	Non-public
National Science Foundation	USA	Public	WHO	–	Public
Fundacao de Amparo a Pesquisa do Estado de Sao Paulo	Brazil	Public	German Research Foundation	Germany	Public
Fundacao Carlos Chagas Filho de Amparo a Pesquisa do Estado do Rio de Janeiro	Brazil	Public	Canadian Institutes of Health Research	Canada	Public
Wellcome Trust	UK	Non-public	Medical Research Council	UK	Public

As to the non-public sector, all organisations/companies in the top ten list are from Europe: GlaxoSmithKline (GSK) and Roche, Inc., and the fourth wealthiest charitable foundation in the world, the Wellcome Trust (2018), which ranked sixth and tenth in supporting Ebola and Zika research, respectively.

**Fig. 11:**
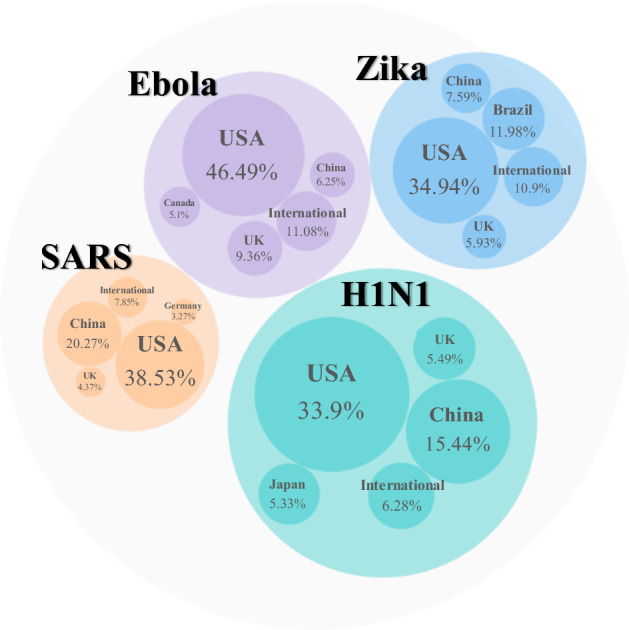
The top 5 countries with the highest proportion of grants on publications of the four infectious diseases

From the perspective of countries and world regions, funding agencies in the USA, China, and the UK contributed most to supporting research in response to public health emergencies, as shown in Fig. 11. In all four cases, the USA ranks first. China is second for SARS and H1N1, and the UK ranks third for SARS and Ebola. Of particular note is that Brazil ranks second in supporting the research on Zika, which is in line with our previous results. Other countries in the top five list are Japan for H1N1, Germany for SARS and Canada for Ebola.

## Preliminary analysis: COVID-19

This ongoing 2019–2020 COVID-19 pandemic has spread globally with unprecedented speed, which raised considerable concern from scientists in various fields from all over the world. The sharply increasing number of papers in the beginning stage of COVID-19 substantiates the immediate and decisive response of academia to public health emergencies again. Perhaps the most remarkable observation, before we examine the research literature on COVID-19 in detail, is how this pandemic has catalysed such intensive scientific activities, which generated an unprecedented amount of knowledge and allowed research to move faster than during any previous outbreak (Kupferschmidt 2020).

Following the retrieval strategies given in Table 3, publications both in international and domestic journals of China were collected on 9 April 2020. The publication data of domestic journals in China was added for two reasons. First, China was one of the first countries on the front line of fighting the pandemic, and, second, China has been acknowledged by the WHO (2020b) as having “rolled out perhaps the most ambitious, agile and aggressive disease containment effort in history” in response to COVID-19, much of which was informed by research findings. Therefore, the studies from China hold a special position that could provide a comprehensive understanding of the efforts made by the whole of Chinese academia.

**Fig. 12:**
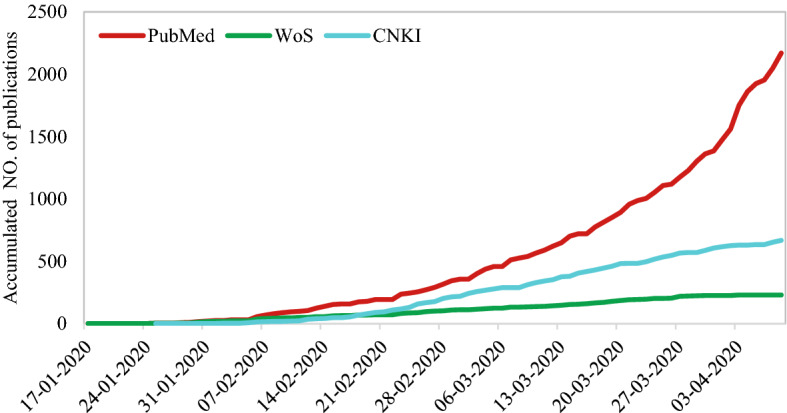
Accumulated number of COVID-19 publications in different databases

Figure 12 shows the cumulative number of publications sourced from each of the different databases from the middle of January to early April. The *Entrez Date*, i.e., the date that the record was added to PubMed, was adopted here to represent the publication time indexed by PubMed.^3^ Due to the incompleteness of ‘Publication Date’ information in WoS, the publication time in WoS was acquired through two approaches: (1) for papers with PubMed ID (PMID), matching papers to PubMed and adopting the *Entrez Date* as publication time, (2) for papers without PMID, searching manually and taking the first online published date as publication time. For publications in CNKI, publication time was directly obtained from the database.

As Fig. 12 shows, PubMed dwarfs CKNI and WoS for the number of publications and growth rate. The low COVID-19 topic coverage in WoS is to a large extent a consequence of the update time of document indexing for the database but also due to the coverage of its journal collection. The first study on COVID-19 indexed by CNKI emerged on 25 Jan 2020, somewhat later than PubMed and WoS. However, the number of papers published in domestic journals still reflects strong growth.

**Fig. 13:**
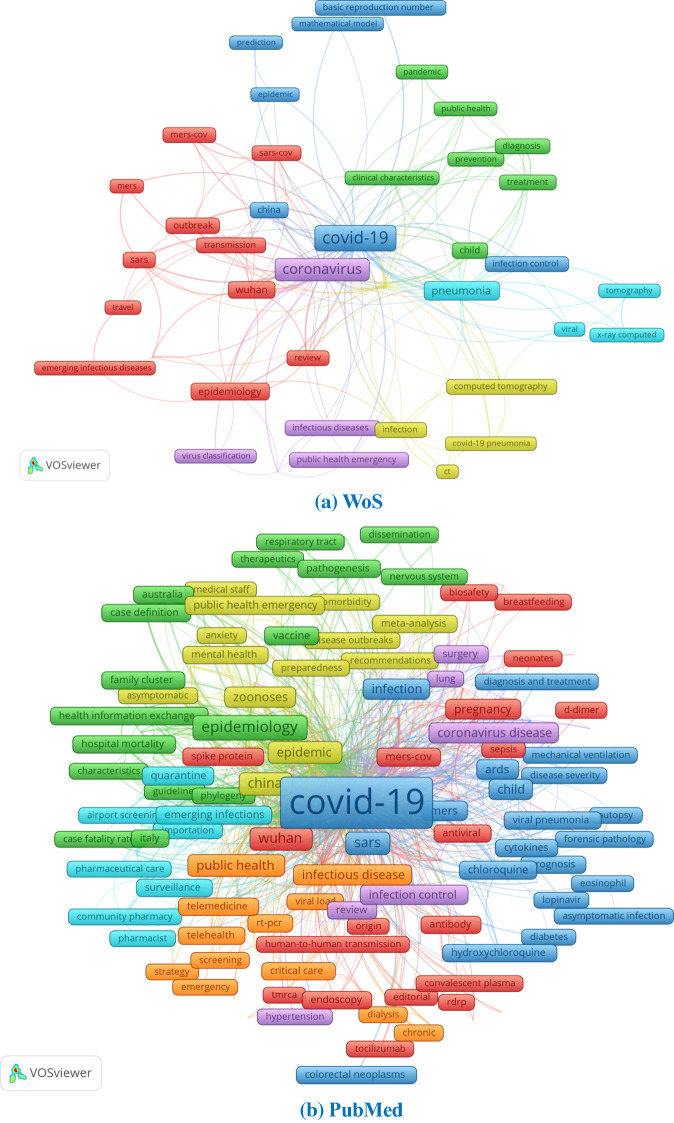

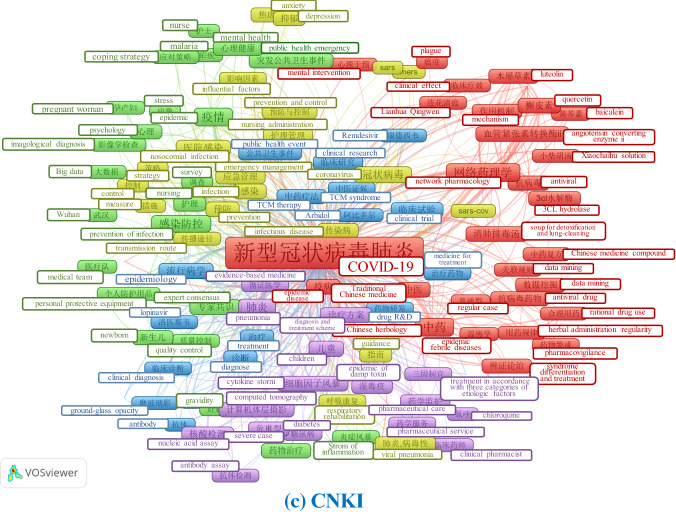
Keywords co-occurrence of COVID-19 publications in different databases using VOSviewer. [*Note* English translations of the Chinese words in (**c**) have been added to the map manually]

The keywords provided with each paper show the different focuses of publications across the databases. We chose three as the threshold of the minimum number of keywords’ occurrence and merged similar terms to generate better visualizations. Figure 13 provides the author-keyword co-occurrence maps using VOSviewer for each database. Apart from the high-frequency terms and phrases related to the name of the virus (e.g., COVID-19, SARS-CoV-2), it is quite apparent that research on other coronavirus diseases, such as “MERS” and “SARS” is the common interest for both papers in the international and domestic journals. With the spread of COVID-19 around the world, epidemic prediction, control and management strategies have also become the general concern of academia. Furthermore, mental health under the crisis time (e.g., anxiety, depression) and pre-existing condition and diseases that may complicate the disease course and cause severe cases of COVID-19 (e.g., old age, obesity, diabetes) also attracted attention for scholars from all over the world.

Publications in international journals reflect the focus on fundamental research on the virus and the development of a vaccine as reflected by keywords like “genome”, “vaccine” etc. The susceptibility of the population, public health concerns and the disease’s status in different countries (e.g., Australia, China) as an epidemic/pandemic also received considerable attention in papers indexed by PubMed. Unlike the publications in international databases, those indexed in the CNKI focus more on clinical studies and diagnosis and treatment schemes. Terms like “clinical trial”, “diagnosis and treatment scheme”, “clinical features”, “Remdesivir” occur frequently. As the core of the outbreak from the end of January until early March, China required timely research on treatment schemes to diagnose patients and prevent further spread. In addition, societal issues such as medical support teams from other provinces in China to Hubei province and personal protective equipment also received attention from research published in domestic journals. It is worth noting that traditional Chinese medicine features prominently in these studies, as evidenced by terms in red clusters, such as “Chinese medicine”, “Chinese herbology”, “syndrome differentiation and treatment” and so forth.

**Fig. 14:**
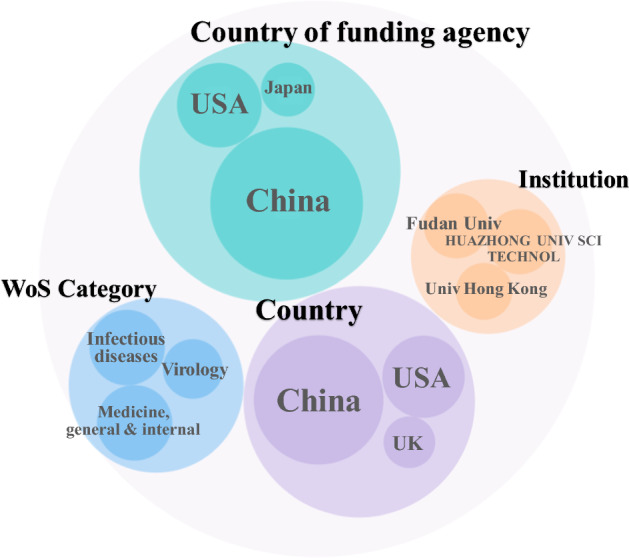
Top three items in institutions, countries, disciplines and countries of funding agency for COVID-19 publications in WoS

Figure 14 depicts the countries, institutions, subjects and countries of funding agencies with the highest number of publications indexed by WoS. We limited this analysis to publications from WoS for comparability with the previous analysis. Chinese scholars have contributed to more than 50% of the papers related to COVID-19, and the majority of the projects have been granted by Chinese funding agencies with the National Natural Science Foundation of China (NSFC) at the top of the list. The three institutions with the highest research output are Chinese universities. In line with previous epidemics, the field with the most studies is infectious diseases.

**Fig. 15:**
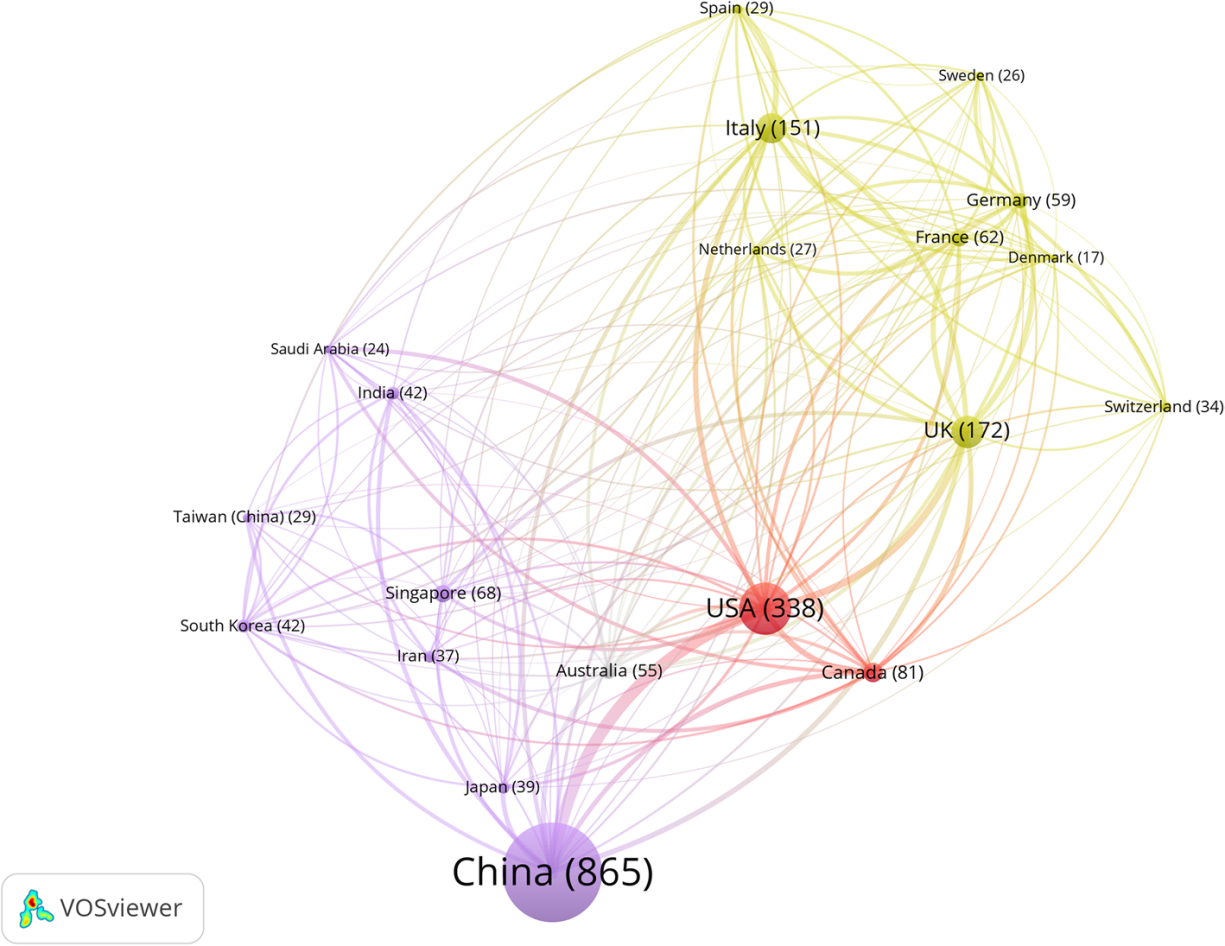
International collaboration pattern of COVID-19 research

To gain insight into how COVID-19 is being studied collaboratively from different countries, we used publications indexed by PubMed to observe the international collaborative pattern due to its relatively high volume of publication data at the present stage. Figure 15 was generated following the same rules as Fig. 4. As Fig. 15 shows, China, as the heart of the outbreak in the initial period, has taken a prominent role in COVID-19 research with the largest number of publications, followed by the USA and the UK. Close collaboration is observed between the two leading countries of China and the USA. Apart from the UK, Italy stands out among other European countries with 151 publications in total. As of 16 April 2020, Italy is one of the world’s centres of the COVID-19 outbreak with 165,155 confirmed cases.^4^

Chinese researchers have consistently played an important role in contributing to knowledge production on emergent epidemics in previous PHEICs since 2000, which signals the growing role of Chinese scholars in international academia. As COVID-19 continues, a vivid public discussion has arisen in China on whether scientific achievements from China should be prioritized to publish in international or domestic journals. Critics of international publication argue that, due to language barrier and possible pay-walls of international journals,  necessary information will not be shared with China in a timely manner with the consequence that gaining and applying knowledge of how to treat the disease and control the epidemic would slow down.

Academia is continuing to adjust its methods to respond outbreaks more efficiently and effectively, which may also result in permanent transformations of scientific and technological systems. Research needs to make more specific and more substantial contributions to the health and wellbeing of people, especially during outbreaks. As Pallari and Lewison (2019) wrote, biomedical research could influence its two main goals: better patient treatment and prevention of illness, by studying the research base of clinical practice guidelines linked to patient treatment, and stories in the mass media as an expression of healthcare policy. In the context of the COVID-19 disease, this means that domestic publication in Chinese could help achieve this goal faster and earlier by reaching the targeted community directly without and paywalls and language barrier that may make relevant literature less accessible to many on the global front line (Larivière et al. 2020).

**Fig. 16:**
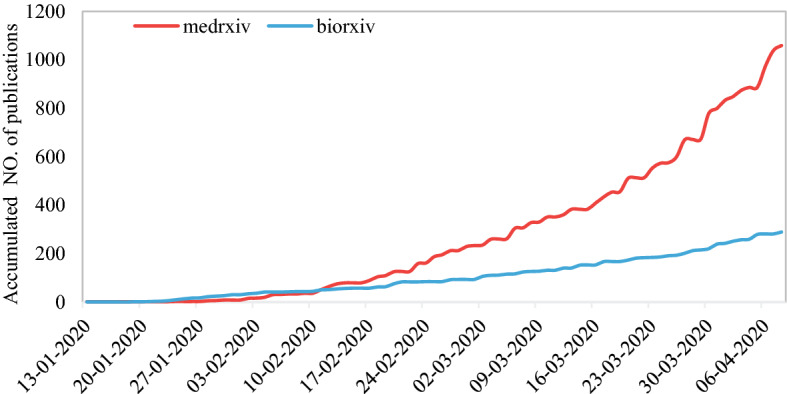
Accumulated number of papers posted on bioRxiv and medRxiv [*Note* Publication data was acquired from https://connect.biorxiv.org/relate/content/181 (COVID-19 SARS-CoV-2 preprints from medRxiv and bioRxiv) on 10 April, 2020**]**

In addition to formally published papers in scholarly journals, many findings related to COVID-19 have been publicly shared as preprints. Preprints have the benefit of accelerating the release of results with a solution that provides fast evidence-based responses, although not peer-reviewed and thus officially unverified (Johansson et al. 2018; Chen et al. 2017). The desire, and perhaps necessity, to publish as preprints have been reflected thoroughly during the COVID-19 outbreak so far. According to Kupferschmidt (2020), the plethora of data has been released daily by preprint servers that did not even exist a decade ago. For example, by early April, two of the largest biomedical preprint servers, bioRxiv and medRxiv, had already posted more than 1300 papers on COVID-19 (see Fig. 16). The two servers “are currently getting around ten papers each day on some aspects of the novel coronavirus,” says John Inglis, head of Cold Spring Harbor Laboratory Press, which runs both servers (Kupferschmidt 2020).

Johansson et al. (2018) found that preprints posted during the Ebola and Zika outbreaks published novel analyses and new data more than 100 days before the publication of the journal version, which is a substantial acceleration of data dissemination and information sharing. However, despite all benefits of preprint publication, there are also some validity issues. Without any peer review or other quality control, there is the risk of disseminating inaccurate results and unvalidated information. A preprint paper (deposited on bioRxiv) stating “uncanny” similarities between SARS-CoV-2 and HIV has fostered conspiracy theories about genetic engineering and might serve as an example. Although the paper was retracted, it still raised a vivid discussion on this issue, leaving people in doubt. Notes have been posted on bioRxiv and medRxiv for each paper to emphasise that preprints only contain preliminary findings. Regardless of whether scholars choose formal publication channels such as a peer-reviewed international or domestic journal or rather publish a preprint, sharing information in a timely manner and open science is of great importance if academia is to build the first line of defence against infectious diseases. With increasing the scale and complexity of the scientific study, a global vision and awareness of international collaborations are essential for scholars to enhance their research ability, the quality and usefulness of outputs and for disseminating their findings in high-level journals. For academia, joint efforts should be made to promote the timely and wide dissemination of relevant information, which is critical for saving lives in times of crisis (Larivière et al. 2020).

## Conclusion and discussion

In this work, we have explored the response patterns of academia to six international public health emergencies in terms of the number of publications, geographic region, subject matter, institutional sector and funding agency. In two separate analyses, we compared academia’s response to five outbreaks of four viruses—Ebola, H1N1, Zika and SARS—with research-activity patterns in the early stages of the COVID-19 pandemic.

Our analysis showed that researchers typically respond quickly to public health emergencies with a sharp increase in the number of publications immediately following a PHEIC by the WHO and during each outbreak. Countries/regions give greater emphasis to epidemics in their own region. However, Europe and North America are also concerned with outbreaks in other, developed and less developed areas through conducting intensive collaborative research with the core countries/regions of the outbreak, such as in the case of Ebola in Africa. As a contrast, research on SARS is primarily conducted by the epicentre of the outbreak—China, with a joint force of USA. The participation of European countries in SARS research is relatively low. In terms of the research field, most papers have been published in the highly relevant disciplines ‘Virology’ and ‘Infectious Diseases’. There are also clear indications that European and American countries pay closer attention to the public health aspects of outbreaks, while China places more emphasis on biochemistry & molecular biology, and Japan tends to focus on pharmacology. Our results also indicate that universities and public funding agencies are the main respondents in global health emergencies.

As the core area of the COVID-19 outbreak in the beginning stage, researchers in China are playing a prominent role in producing knowledge and international dissemination of scientific information regarding the virus and the disease. While preprint proved important means of extremely fast response and scholarly communication, the lack of peer-reviewing also raises the risk of spreading inaccurate information. More research on preprint publishing is needed to investigate how preprints might affect the response patterns of academia to health emergencies.

Outbreaks of epidemics on that scale may also affect or even result in transformations of national health security systems. After the SARS outbreak in 2003, the Chinese government undertook major programs to strengthen the public health systems (Wu and Ye 2019). Despite these initiatives, however, the attention and efforts on improving the system are declining over time. Some argued that severe shortcomings of public health and security system had been revealed in this outbreak (Ding et al. 2020). Sustained attention from both governments and academia is necessary if treatments, cures and preventative measures have to be developed for diseases caused by these viruses and their possible future mutations. Recent public health emergencies have uncovered the disruptive effect of outbreaks on individuals and society, leading many scholars and practitioners to call for increased investments in “global health security” (Puyvallée et al. 2018). Their implementation in system reforms could be an interesting topic for future research.

## Footnotes

1 https://www.who.int/ihr/procedures/pheic/en/.

2 https://www.nlm.nih.gov/mesh/intro_retrieval.html.

3 https://www.nlm.nih.gov/bsd/mms/medlineelements.html#edat.

4 https://coronavirus.jhu.edu/map.html
